# Genome-Wide DNA Methylation and Its Effect on Gene Expression During Subclinical Mastitis in Water Buffalo

**DOI:** 10.3389/fgene.2022.828292

**Published:** 2022-03-15

**Authors:** Varij Nayan, Kalpana Singh, Mir Asif Iquebal, Sarika Jaiswal, Anuradha Bhardwaj, Chhama Singh, Tanvi Bhatia, Sunil Kumar, Rakshita Singh, M. N. Swaroop, Rajesh Kumar, S. K. Phulia, Anurag Bharadwaj, T. K. Datta, Anil Rai, Dinesh Kumar

**Affiliations:** ^1^ ICAR-Central Institute for Research on Buffaloes, Hisar, India; ^2^ Centre for Agricultural Bioinformatics, ICAR-Indian Agricultural Statistical Research Institute, New Delhi, India; ^3^ ICAR-National Research Centre on Equines, Hisar, India

**Keywords:** water buffalo, subclinical mastitis, DNA methylation, gene expression, web resource

## Abstract

Subclinical mastitis (SCM) in buffalo is one of the most challenging paradoxes for the dairy sector with very significant milk production losses and poses an imminent danger to milch animal’s milk-producing ability. We present here the genome-wide methylation specific to SCM in water buffalo and its consequential effect on the gene expression landscape for the first time. Whole-genome DNA methylation profiles from peripheral blood lymphocytes and gene expression profiles from milk somatic cells of healthy and SCM cases were catalogued from the MeDIP-Seq and RNA-Seq data. The average methylation in healthy buffaloes was found to be higher than that in the SCM-infected buffaloes. DNA methylation was abundant in the intergenic region followed by the intronic region in both healthy control and SCM groups. A total of 3,950 differentially methylated regions (DMRs) were identified and annotated to 370 differentially methylated genes (DMGs), most of which were enriched in the promoter region. Several important pathways were activated due to hypomethylation and belonged to the *Staphylococcus aureus* infection, Th17 cell differentiation, and antigen processing and presentation pathways along with others of defense responses. DNA methylome was compared with transcriptome to understand the regulatory role of DNA methylation on gene expression specific to SCM in buffaloes. A total of 4,778 significant differentially expressed genes (DEGs) were extracted in response to SCM, out of which 67 DMGs were also found to be differentially expressed, suggesting that during SCM, DNA methylation could be one of the epigenetic regulatory mechanisms of gene expression. Genes like CSF2RB, LOC102408349, C3 and PZP like, and CPAMD8 were found to be downregulated in our study, which are known to be involved in the immune response to SCM. Association of DNA methylation with transposable elements, miRNAs, and lncRNAs was also studied. The present study reports a buffalo SCM web resource (BSCM2TDb) available at http://webtom.cabgrid.res.in/BSCM2TDb that catalogues all the mastitis-related information of the analyses results of this study in a single place. This will be of immense use to buffalo researchers to understand the host–pathogen interaction involving SCM, which is required in endeavors of mastitis control and management.

## Introduction

Water buffalo (*Bubalus bubalis*) has proven to be the “bank on hooves” by reshaping the landscape of agrarian livelihood in South and Southeast Asian Countries. In India, buffaloes contributed 49% (35% indigenous buffaloes and 14% nondescriptive buffaloes) towards the total milk pool amounting to a massive 91.817 million tonnes of milk. A whopping 44.76 million buffaloes were in milk with an average yield of 5.62 kg/day pan-India in the year 2018–2019 (Basic Animal Husbandry Statistics, 2019). However, mastitis remained a major constraint with huge production and economic losses. Mastitis is considered to be one of the expensive diseases affecting dairy cattle worldwide in terms of production losses (Sinha et al., 2014; Ruegg and Erskine, 2015; Beniæ et al., 2018). Mastitis is caused by varied pathogens leading to the development of subclinical/chronic (25%–65%) or clinical (∼5%) infections (Dufour et al., 2012; AHDB, 2016; Wang et al., 2020) worldwide. The economic loss caused by subclinical mastitis (SCM) is often greater than that caused by clinical mastitis (Kirkpatrick and Olson, 2015). SCM is the inflammation of the mammary gland that does not create visible changes in the milk or the udder. It affects the dairy industry by reducing milk production, decreasing milk quality, and suppressing reproductive performance (Khan and Khan, 2006; Ahmad et al., 2011). An estimated loss of more than $1 billion per year was reported by the United States dairy industry in 1999 (Ott, 1999), increasing to $2 billion per year by 2006. The loss was estimated to be 48€/1,000 L in Ireland (Geary et al., 2013). In India, the annual loss due to mastitis has been estimated to the tune of Rs. 71,651.5 million per year (Sudhan and Sharma, 2010; Rao et al., 2017). Apart from economic losses, SCM has distinct importance in public health due to the risk of antibiotic resistance by consumption of milk with antibiotic residues accumulated due to the indiscriminate use of antibiotics (De Vliegher et al., 2012) for SCM treatment.

*Staphylococcus aureus* is the major cause of SCM in dairy cattle, which causes asymptomatic, persistent, antibiotic-resistant, and reoccurring infections (Song et al., 2016). SCM is caused by a variety of pathogens that can establish chronic infections and include *Escherichia coli*, *Pseudomonas aeruginosa*, *P. mendocina*, *S. chromogenes*, *S. epidermidis*, *Bacillus cereus*, *Klebsiella pneumoniae*, and *Shigella flexneri* (Hoque et al., 2015; Hoque et al., 2020). Similar causal organisms were also reported in buffalo mastitis (Fagiolo and Lai, 2007).

Previous studies showed the role of epigenetics in influencing traits related to health, growth, production, and development in cattle (Ibeagha-Awemu and Zhao, 2015; Reynolds et al., 2017; Sun et al., 2019). The potential contribution of epigenetic regulation in mechanisms of mastitis infection development, especially the role of DNA methylation in the regulation of mammary gland health, has been reported in the case of cattle (Song et al., 2016; Ju et al., 2020; Wang et al., 2020; Zhou et al., 2020). No such study has been reported in water buffalo. There is no web genomic resource for water buffalo mastitis disease with a list of differentially expressed genes (DEGs) and differentially methylated genes (DMGs) to help in understanding the molecular events.

DNA methylation is an important epigenetic regulator of gene expression and chromatin structure (Niazi et al., 2016) that provides stability to the genome by methylation of transposable elements (TEs). DNA methylation is catalyzed by a family of DNA methyltransferases (Dnmts) that forms 5-methylcytosine (5-mC) (Ruzov et al., 2011). Current genome-scale approaches for the determination of DNA methylation are largely based on the detection of 5-mC. MeDIP-Seq is a popular enrichment technique for the methylation status of cytosines captured by noncovalent bonding of 5-mC and antibodies (Taiwo et al., 2012; Li et al., 2015a). It has been used in numerous studies including the first mammalian methylome (Down et al., 2008) and the first cancer methylome (Feber et al., 2011).

Owing to the importance of water buffalo and losses caused by SCM, the present study focuses on the extraction of DMGs from whole-genome methylome (MeDIP-Seq) analysis and DEGs from transcriptome (RNA-Seq) analysis to understand the molecular mechanism of epigenetic regulation of gene expression involving DNA methylation specific to SCM in water buffalo. The present study also explores the association of DNA methylation with TEs, miRNAs, and lncRNAs. All the findings of the study are provided in the form of a user-friendly web resource, Buffalo Subclinical Mastitis Methylome–Transcriptome database (BSCM2TDb), available at http://webtom.cabgrid.res.in/BSCM2TDb. The present study is the first whole-genome methylome study specific to SCM in water buffalo of Murrah breed. The findings of this study can be used to understand the molecular regulation of mastitis disease as well as to identify candidate epigenetic markers related to the disease. It will help buffalo breeders in breed improvement and disease management programs.

## Materials and Methods

### Ethics Statement

This study was approved by the Institute Animal Ethics Committee of the ICAR-Central Institute for Research on Buffaloes (ICAR-CIRB), Hisar.

### Determination of SCM in Buffaloes

A total of 138 lactating Indian Murrah breed water buffaloes from the ICAR-CIRB animal farm (coordinates: 29º10′49.40″N, 75º42′24.87″E) were screened for the incidence of SCM. The milk samples were collected aseptically, noninvasively, and during the normal milking process. Milk was collected under clean and sterile conditions. After the initial milk from the teats was ignored, milk was aseptically collected in sterile containers without touching the container. The sample containers were clearly labeled as fore right (FR), fore left (FL), rear left (RL), and rear right (RR), bearing the animal numbers.

For ascertaining the cases of SCM, the criteria provided by the International Dairy Federation were adopted, and milk samples were subjected to somatic cell count (SCC), bacteriological examination, and antimicrobial sensitivity testing. The milk samples were tested by the cow-side test of the California Mastitis Test (CMT). A total of eight CMT-positive cases for SCM were found. The milk samples found positive with CMT were subjected to SCCs, bacteriological culture, and sensitivity tests for ascertaining the confirmed SCM cases. SCCs were done for the CMT-positive samples and the healthy controls as well, using Newman’s stain by adopting the method given by Schalm et al. (1971). SCC of positive cases found was done by making slides and counting manually. Cases with SCCs of >5 × 10^5^ cells/ml were designated as subclinical. For all positive cases with CMT, 10 ml milk samples were immediately sent for microbiological culture at the Department of Veterinary Microbiology, LUVAS, Hisar, India, for confirmation of mastitis, identification of microorganisms, and susceptibility to different antibiotics. For bacteriological studies, the milk samples were streaked on both 5% sheep blood agar and MacConkey’s lactose agar plates and kept for incubation at 37°C for 24–48 h. The bacterial colonies were further subcultured on blood agar plates and identified by Gram’s staining based on bacterial morphology and colony characteristics. Furthermore, the disc diffusion method (Bauer et al., 1966) was used for antimicrobial sensitivity test based on the zone-size interpretation chart and categorized as sensitive, intermediate, and resistant. The results were recorded for the antimicrobials, namely, enrofloxacin, penicillin G, streptomycin, amoxicillin, oxytetracycline, chloramphenicol, moxifloxacin, levofloxacin, ampicillin, gentamicin, neomycin, amikacin, cloxacillin, and cefoperazone.

### Sample Collection, DNA Isolation, and Preparation of MeDIP-Seq Libraries

The blood samples were collected by jugular venipuncture from the same five SCM-infected lactating Murrah buffaloes (SCM 1–5) and six healthy Murrah buffaloes (C1–C6) as control in EDTA containing blood collection tubes. Furthermore, DNA isolation and purification were performed using the QIAamp blood DNA mini kit (Qiagen) in all samples from DNA Xperts Private Limited, Noida, India, utilizing the protocol adapted by Li et al. (2010) with few modifications and followed for preparing MeDIP-Seq libraries. Library preparation was done by using the NEBNext^®^ Enzymatic Methyl-Seq Kit, followed by immunoprecipitation enriched for methylated DNA fragments using MeDIP buffer and 5-mC-specific monoclonal antibody. It was followed by a quality check by loading 1 µl of sample on an Agilent Technologies 2100 Bioanalyzer using a DNA-specific chip. Finally, 11 MeDIP-Seq libraries were obtained using the Illumina HiSeq 2500 instrument (Illumina Inc., United States) with 2× 50 bp paired-end (PE) sequencing.

### Sample Collection, RNA Isolation, and Preparation of RNA-Seq Libraries

For RNA-Seq libraries, the milk samples were collected from six SCM-infected (SCM 1–6) udder quarters of five SCM-infected Murrah buffaloes and six healthy (C1–C6) udder quarters from six healthy Murrah buffaloes. These samples were centrifuged in 50 ml tubes at 1,500 × *g* for 20 min at 4°C for total somatic cell isolation and later preserved. RNA was isolated by DNA Xperts Private Limited, Noida, India, using the QIAamp RNA blood mini kit (Qiagen) from suspended somatic cells. Furthermore, quality was checked using the Agilent 2100 Bioanalyzer, and electrophoresis was performed on formaldehyde with 1% agarose gel. The polyA-containing mRNA molecules were purified using oligo-dT attached magnetic beads. Finally, cDNA libraries were prepared as per the Illumina TruSeq RNA library preparation protocol (Illumina Inc., United States) and sequenced using the Illumina HiSeq 2500 instrument to obtain 12 RNA-Seq libraries (2× 100 bp PE).

### MeDIP-Seq Data Analysis

Raw PE reads of MeDIP-Seq libraries were passed through a quality check using a FASTQC at Phred score ≥30 and GC distribution >40% and adapter trimming. The processed PE reads were aligned with the buffalo reference genome (GCF_003121395.1 available at https://www.ncbi.nlm.nih.gov/assembly/GCF_003121395.1/) using Bowtie 2 (Langmead and Salzberg, 2012). These aligned reads were used for the correlation analysis using deepTools (Ramirez et al., 2014) and identification of methylated peaks using MACS2 (Zhang et al., 2008) with a *p*-value of 0.01 in all the libraries. The extracted methylated peaks were used for genomic annotation of methylated peaks into genomic regions such as intron, exon, promoter (−1 kb to +100 bp), transcription termination sites (TTS) (−100 bp to +1 kb), and intergenic regions using HOMER (Heinz et al., 2010) utilizing a buffalo RefSeq annotation file. The DMRs in the SCM group were compared to those of the control group using diffReps (Shen et al., 2013) at a *p*-value of 0.001 and |Log2FC| ≥2. Later, DMGs were extracted from DMRs utilizing the buffalo RefSeq annotation file by the Perl script. Functional annotation of DMGs was performed by gene ontology (GO) analysis using BLAST2GO (Conesa and Götz, 2008), and KEGG pathway analysis was performed using the clusterProfiler package (Yu et al., 2012) of Bioconductor for DMGs at a *p*-value <0.1.

### RNA-Seq Data Analysis

For RNA-Seq data analysis, all 12 RNA-Seq data libraries were passed through quality check by FASTQC v0.11.5 (Andrews, 2010) using the parameters of a Phred score ≥20 and GC distribution >40%. The bases with a <20 Phred score were trimmed using FASTX-Toolkit v0.0.14 (https://github.com/agordon/fastx_toolkit). This was followed by transcriptome assembly using Trinity v2.2.0 (Grabherr et al., 2011). The differentially expressed regions (DERs) were extracted using DESeq2 of the R package v1.26.0 (Love et al., 2014). For the extraction of DEGs, open reading frames (ORFs) were predicted using TransDecoder v5.5.0 (Haas and Papanicolaou, 2019), and annotation was performed using Trinotate v3.2.0 at |log_2_FC| ≥03 and FDR <0.05 (Bryant et al., 2017). Alignment of DMGs with the buffalo reference genome was performed to extract the coordinate information using BLAST with a parameter e-value of 1e−30 using the Perl script. Finally, the in-house Perl scripts were used to get buffalo gene IDs to compare with DMGs with the help of the coordinate information within the buffalo RefSeq annotation file.

**FIGURE 1:**
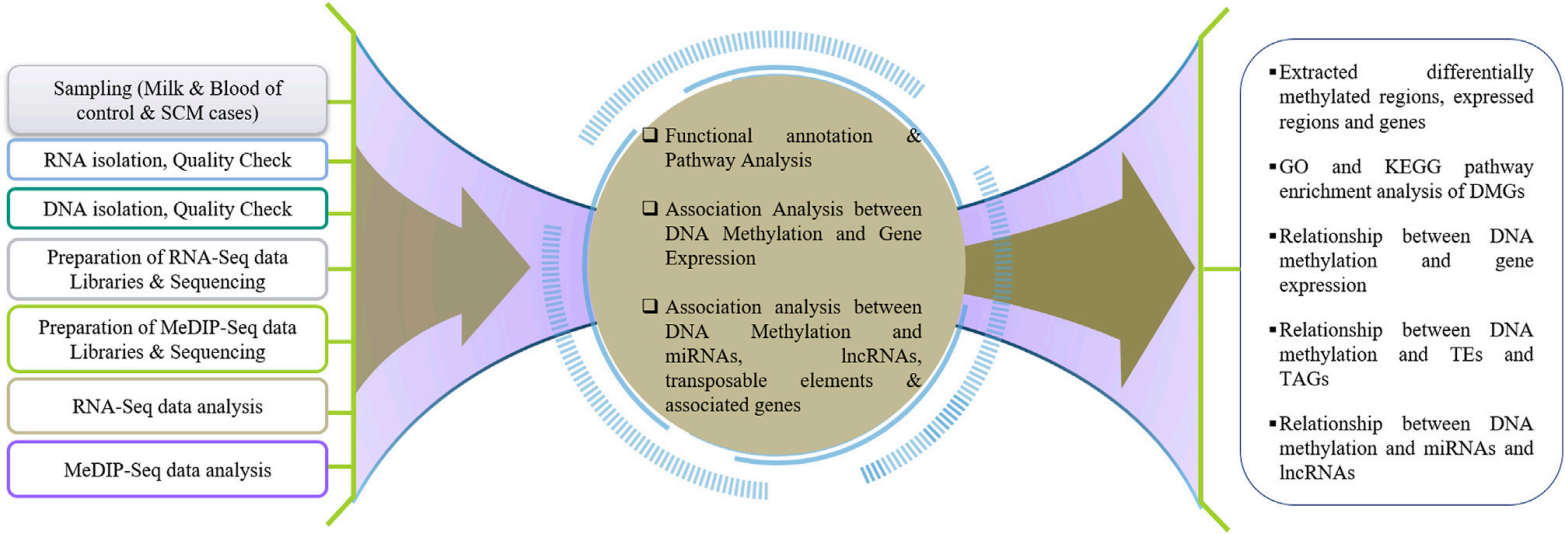
Schematic workflow towards understanding the genome-wide DNA methylation and its effect on gene expression during SCM in water buffalo.

To understand the effect of DNA methylation on gene regulation during SCM in buffalo, the results of DNA methylation analysis (MeDIP-Seq) were compared with the results of transcriptome analysis (RNA-Seq). The DMGs and DEGs were compared to find the genes repressed by hypermethylation and expressed by hypomethylation, i.e., a negative relationship as reported by Lou et al. (2014) in the promoter and TTS regions, while a positive relationship in case of exon and intron as reported by Suzuki and Bird (2008), Ball et al. (2009), and Lev Maor et al. (2015). Figure 1 represents the pipeline for understanding genome-wide DNA methylation and its effect on gene expression during SCM in buffalo.

### Association of Methylation with TEs, lncRNAs, and miRNAs

In order to study the role of DNA methylation in genome stability, TEs were searched, which were found to overlap with DMRs using Censor (Huda and Jordan, 2009). The probable TE-associated genes (TAGs) were also extracted by comparing TEs with the buffalo RefSeq annotation file using the Perl script. Furthermore, pre-miRNA sequences of known buffalo miRNAs were mapped with DMRs using BLAST (Altschul et al., 1990) to see the methylation in putative miRNA genes, which were termed as methylation-regulated miRNA genes. Methylation-regulated miRNAs were used to study their target mRNAs from DEGs specific to SCM by using psRNATarget (Dai et al., 2018) to see the consequent indirect effect of DNA methylation through the regulation of miRNA expression. The psRNATarget also predicts the mode of action of miRNAs on their target such as cleavage or binding, which disables their targets for further action. Moreover, functional annotation showed that a few DMGs were encoding lncRNAs, which were considered methylated lncRNA genes, and their respective lncRNAs were termed as methylation-regulated lncRNAs. Targets of methylation-regulated lncRNAs were identified using LncTar (Li et al., 2015b) to see the consequent indirect effect to DNA methylation through regulation of lncRNA expression.

### Development of the Web Resource, BSCM2TDb

BSCM2TDb is a three-tier architecture-based relational database, freely accessible at http://webtom.cabgrid.res.in/BSCM2TDb/. All the analyses results like DMRs, DMGs, DM-lncRNAs (methylation-regulated lncRNAs), DM-miRNAs (methylation-regulated miRNAs), DM-TEs, and DM-TAGs from MeDIP-Seq data analysis along with DEGs and DM-DEGs from RNA-Seq data analysis were catalogued and stored in the backend in a MySQL database. The web interface was developed in PHP and launched by the Apache 2 server. BSCM2TDb contains tabs, *namely*, Home, Statistics, Data, Tutorial, and Team.

## Results

### MeDIP-Seq Data Analysis

A total of ∼745 million reads were found in the MeDIP-Seq libraries with an average of ∼67.7 million reads in each library. A total of 33.8 million PE reads passed through quality check with an average of 47% GC content (Supplementary Table S1). An average of ∼71% alignment was attained for all libraries with the buffalo RefSeq genome. Pearson’s correlation coefficients among all the libraries were in the range of 0.71–0.99 (Supplementary Figure S1).

### Peak Calling and their Genomic Annotation

A total of 189,474 peaks were extracted from the control group with an average of 31,579 peaks, while 154,803 peaks were extracted from the SCM group with an average of 30,960 peaks (Supplementary Table S2). The result of genomic annotation of these peaks showed that the maximum number of peaks belonged to the intergenic region (75%–76%), followed by the intron (∼14%), promoter (∼5%), TTS (∼4%), and exon (<1%) (Supplementary Figure S2).

### Identification of DMRs and DMGs

**FIGURE 2:**
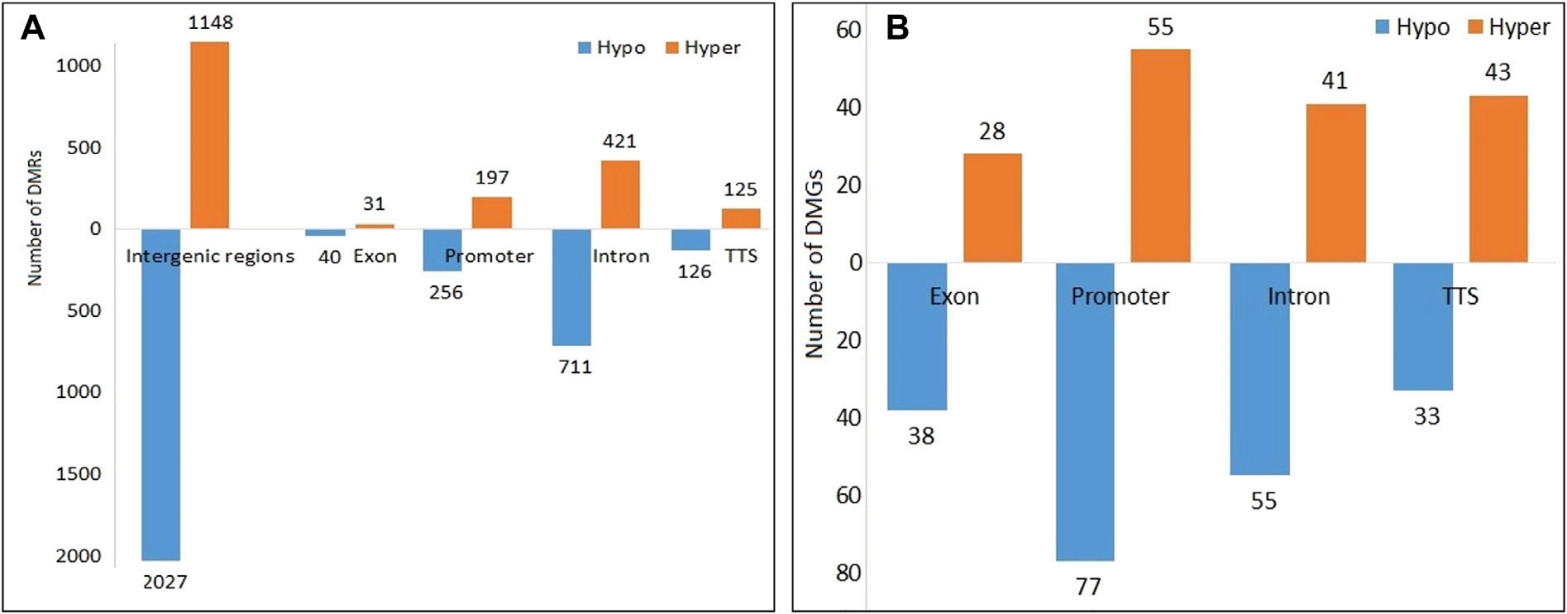
Hypomethylated and hypermethylated **(A)** DMRs and **(B)** DMGs in the SCM group in comparison to the control group.

A total of 3,950 DMRs were extracted from the peaks of the SCM group with respect to peaks of the control group, out of which 2,451 DMRs were hypomethylated while the remaining 1,449 were hypermethylated. The distribution of methylation counts in the form of histograms is shown in a Circos diagram (Supplementary Figure S3) found within DMRs for the control and SCM groups in all 25 chromosomes. Out of 3,950 DMRs, 71, 1,132, 2,043, 251, and 453 DMRs belonged to exon, intron, intergenic regions, TTS, and promoter regions, respectively. Figure 2A shows hypomethylated and hypermethylated DMRs within various genomic regions.

A total of 370 DMGs were extracted from 3,950 DMRs, out of which 169 DMGs were hypermethylated and 201 DMGs were hypomethylated. Genomic distribution of 370 DMGs showed that 66, 132, 96, and 76 DMGs belonged to exons, promoters, introns, and TTS regions, respectively (Figure 2B). Functionally, it was found that out of these 370 DMGs, 140, 217, and 1 DMGs were encoding for lncRNAs, proteins, and snRNA, respectively, along with the remaining 12 pseudogenes.

### GO and KEGG Pathway Enrichment Analyses of DMGs

**FIGURE 3:**
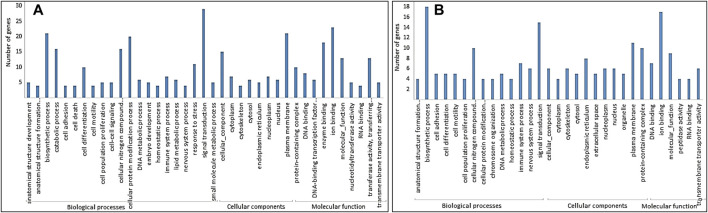
GO terms in three classes: biological processes, cellular components, and molecular function for **(A)** hypomethylated DMGs and **(B)** hypermethylated DMGs.

The GO analysis of DMGs showed that the biological process was the largest class in both hypomethylated and hypermethylated DMGs. Molecular function was the least abundant in hypermethylated DMGs, while cellular component was the least abundant in hypomethylated DMGs (Figures 3A,B). More biological processes were enriched with hypomethylated DMGs than with hypermethylated DMGs. The most enriched GO terms in hypermethylated DMGs in decreasing order were biosynthetic process, ion binding, signal transduction, cellular nitrogen compound, plasma membrane, and protein-containing complex. The most enriched GO terms in hypomethylated DMGs in decreasing order were signal transduction, ion binding, biosynthetic process, plasma membrane, cellular protein modification process, enzyme binding, catalytic process, cellular nitrogen compound, cellular function, and transferase activity.

**FIGURE 4:**
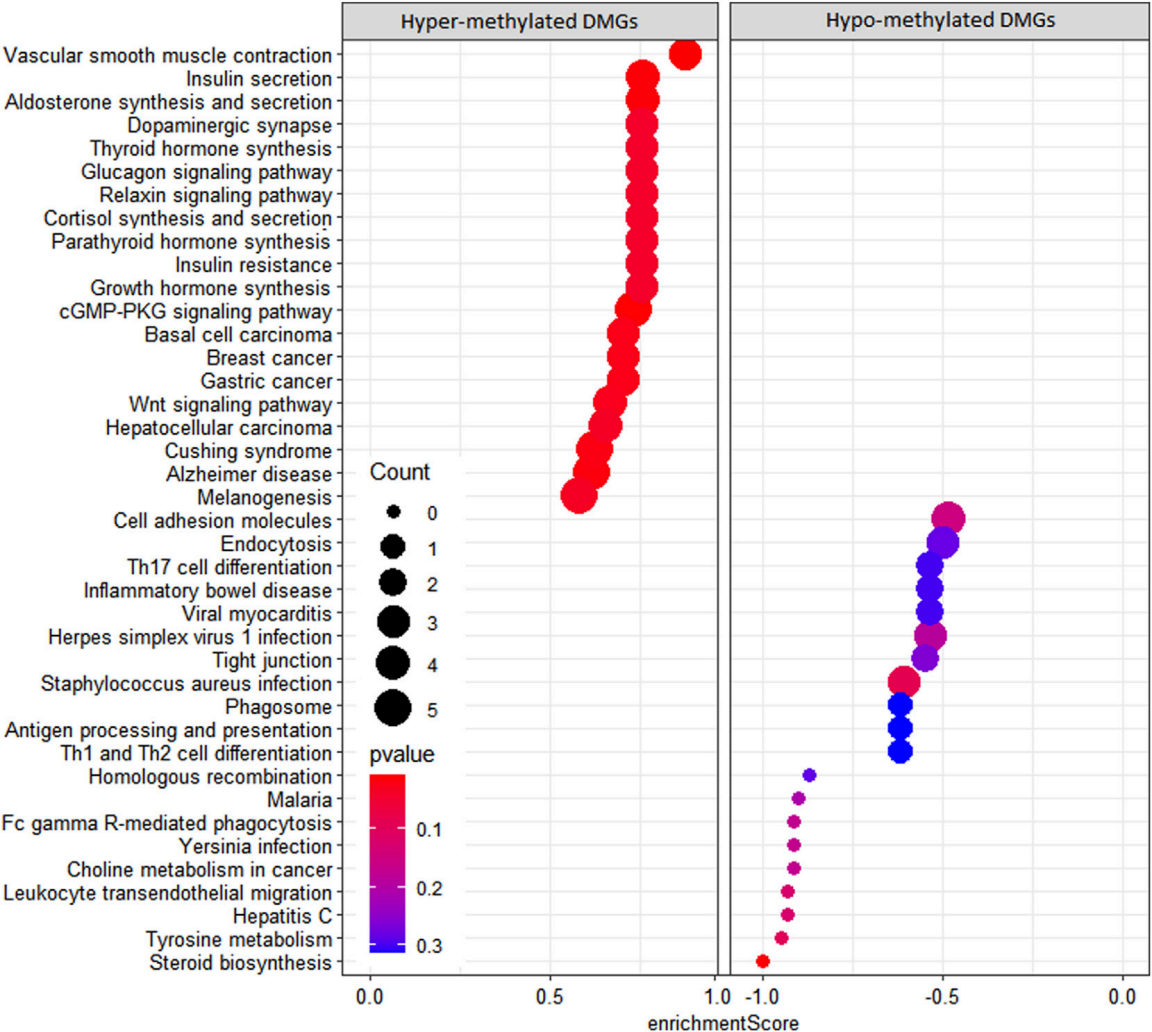
Dot plot of KEGG pathways enriched by hypermethylated and hypomethylated DMGs.

**FIGURE 5:**
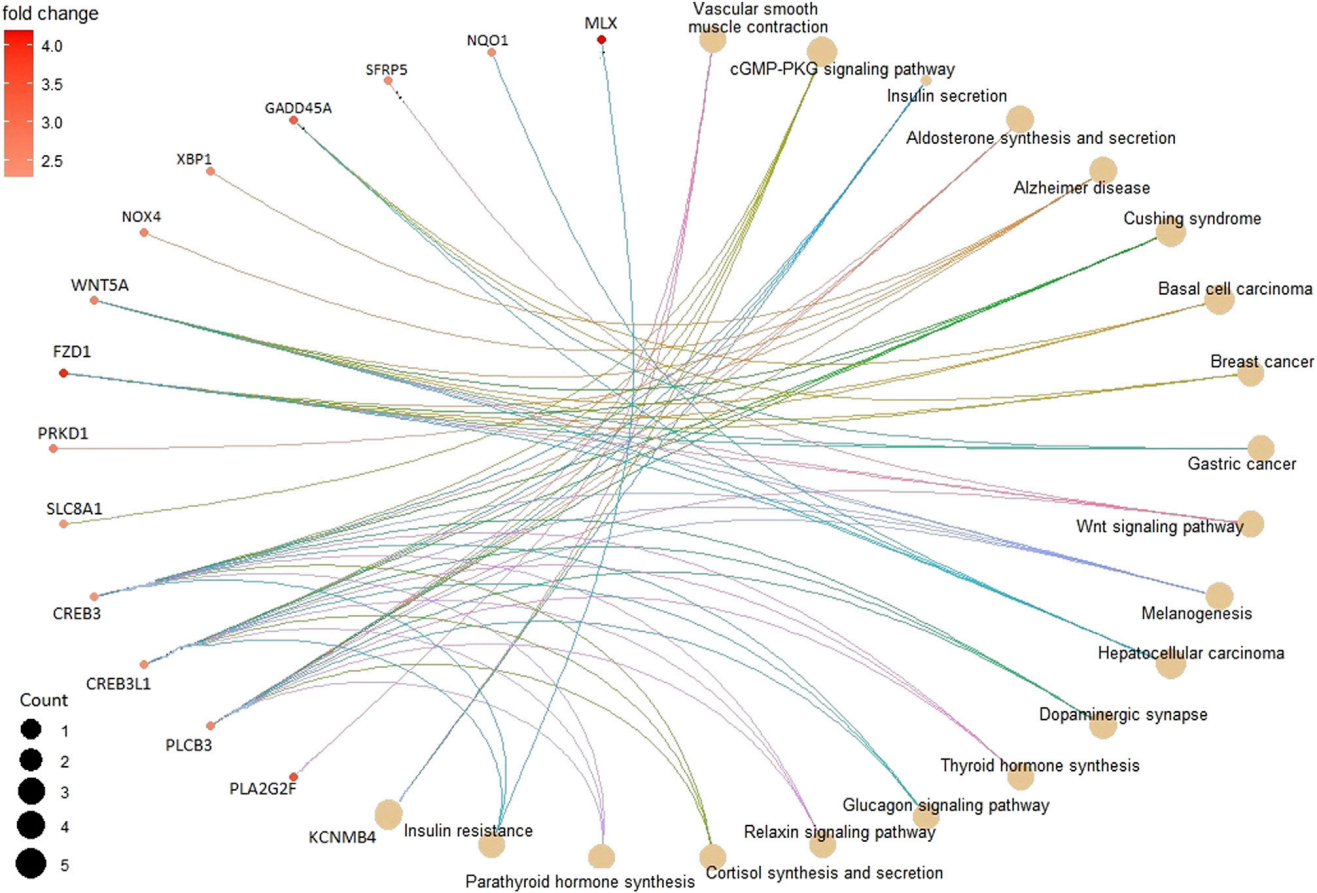
Pathway network showing the 14 most enriched DMGs (in smaller circles) connected to 20 enriched pathways (in bigger brown circles) depicting their relation.

In the KEGG pathway enrichment analysis, 142 pathways were found for 367 DMGs at a *p*-value <0.1, out of which 40 most enriched pathways were represented by hypomethylated and hypermethylated DMGs (20 each) as shown in the form of a dot plot (Figure 4). The network of pathways showed the 14 most enriched DMGs involved in 20 enriched pathways (Figure 5). Phospholipase C beta 3 (PLCB3):102413748 was found to be connected to 13 pathways, cAMP-responsive element-binding protein 3 like 1 (CREB3L1):102406078 and (CREB3):102395032 both connected to 12 pathways, frizzled-1-like (LOC102414897):102414897 connected to eight pathways, Wnt family member 5A (WNT5A):102403744 connected to eight pathways, and growth arrest and DNA damage-inducible alpha (GADD45A):102401934 connected to four pathways, which were the most enriched DMGs in the top networks (Figure 5).

### RNA-Seq Data Analysis and Identified DEGs

In all 12 RNA-Seq libraries, a total of ∼358 million PE reads were found with an average of ∼15 million PE reads, which were aligned with an average of 75.65% (Supplementary Table S1). Furthermore, a total of 21,028 DERs were extracted from the SCM group with respect to the control group, out of which 8,408 and 12,620 DERs were downregulated and upregulated, respectively. While annotating these DERs with the buffalo RefSeq annotation file, 4,778 DEGs were extracted, out of which 2,908 and 1,870 DEGs were downregulated and upregulated, respectively.

### Association of DNA Methylation with Gene Expression

A comparative study of DMGs from MeDIP-Seq analysis and DEGs from RNA-Seq analysis showed a total of 67 DMGs to have differential methylation along with differential expression. Their level of methylation along with the level of expression is shown in Supplementary Figure S4. Out of 67 DMGs, 33 (49.25%) were found to have gene expression as per methylation; i.e., 25 DMGs were negatively correlated with gene expression having methylation in promoter and TTS regions and the remaining eight DMGs were positively correlated with gene expression having methylation in genic regions (exon and intron) (Tables 1 and 2).

**TABLE 1:** Hypomethylated and hypermethylated DMGs and their expression due to methylation in different genomic regions.

DNA methylation/gene expression	Exon	Promoter	Intron	TTS
Hypomethylated/downregulated	2	9	0	4
Hypermethylated/upregulated	2	2	4	8
Hypomethylated/upregulated	3	9	3	1
Hypermethylated/downregulated	5	10	0	5

**TABLE 2:** Coordinated relation of DMGs with DNA methylation and gene expression according to genomic regions.

DNA methylation/gene expression	Gene IDs and their protein product
Hypo/Down in exon	espin-like (ESPNL), probable phospholipid-transporting ATPase IA-like (LOC102398473)
Hypo/Up in promoter	MAF bZIP transcription factor K (MAFK), selectin E (SELE), cryptochrome circadian clock 1 (CRY1), plastin 3 (PLS3), StAR-related lipid transfer domain containing 10 (STARD10), zinc finger protein 684 (ZNF684), calcium voltage-gated channel auxiliary subunit beta 2 (CACNB2), AT-rich interaction domain 5A (ARID5A), proline rich 15 like (PRR15L)
Hypo/Up in TTS	phosphatidylinositol-4-phosphate 5-kinase type 1 beta (PIP5K1B)
Hyper/Down in promoter	mediator complex subunit 25 (MED25), biogenesis of lysosomal organelles complex 1 subunit 6 (BLOC1S6), cAMP responsive element binding protein 3 like 1 (CREB3L1), FRY microtubule binding protein (FRY), protein kinase C-binding protein 1-like (LOC102401961), VPS11, CORVET/HOPS core subunit (VPS11), colony stimulating factor 2 receptor beta common subunit (CSF2RB), granulocyte-macrophage colony-stimulating factor receptor subunit alpha-like (LOC102408349), C3 and PZP like, alpha-2-macroglobulin domain containing 8 (CPAMD8), Myb like, SWIRM and MPN domains 1 (MYSM1)
Hyper/Down in TTS	TBC1 domain family member 9B (TBC1D9B), uncharacterized (LOC102390914), ARP8 actin-related protein 8 homolog (ACTR8), zinc finger and BTB domain containing 20 (ZBTB20), DDB1 and CUL4 associated factor 6 (DCAF6)
Hyper/Up in exon	Glycosyltransferase 1 domain containing 1 (GLT1D1)
Hyper/Up in intron	Uncharacterized (LOC102400551), uncharacterized (LOC102406144)

### Association of DNA Methylation with TEs and TAGs

A total of 3,377 TEs were found within DMRs, out of which 949 TEs belonged to introns, 52 (44 hypomethylated and 8 hypermethylated) to exons, 336 (241 hypomethylated and 95 hypermethylated) to promoters, 155 (109 hypomethylated and 46 hypermethylated) to TTS, and 1,896 to intergenic regions. These TEs belonged to eight major classes and their included subclasses along with their frequencies (Table 3) such as (1) DNA 1,022; (2) ERV 445; (3) integrated virus 39; (4) interspersed repeat 35; (5) LTR 570; (6) multicopy genes 6; (7) non-LTR 2,204; and (8) simple 5. The largest class found was non-LTR, and the largest subclass found was SINE, followed by LTR, Gypsy, hAT, RTE, and ERV3.

**TABLE 3:** TEs in DMRs categorized into classes and their subclasses along with their frequencies.

S. No	Class and number of TEs	Subclasses of TEs and their frequencies
1	DNA: 1,022	DNA-68, Academ-11, Crypton-17, Dada-8, Enspm/CACTA-91, Ginger-7, Harbinger-64, hAT-306, Helitron-87, IS3EU-4, ISL2EU-14, Kolok-16, Mariner-129, Merlin-4, MuDR-82, P-11, Novosib-4, PiggyBac-19, Polinton-44, Sola-24, Transib-7, Zator-2, Zisupton-3
2	ERV: 445	ERV-17, ERV1-152, ERV2-29, ERV3-246, ERV4-1
3	Integrated virus: 39	Caulimovirus-29, DNAV-10
4	Interspersed repeat: 35	-
5	LTR: 570	LTR-13, BEL-24, Copia-160, DIRS-19, Gypsy-354
6	Multicopy gene: 6	Multicopy_gene-4, rRNA-2
7	Non-LTR: 2,204	Non-LTR-33, L1-517, L2-30, R1-13, R2-1, R4-5, Rex1-4, Loa-6, NeSL-1, CR1-198, Crack-5, CRE-4, Daphne-20, Hero-5, I-6, Ingi-1, Jockey-14, Kiri-6, Nimb-11, Outcast-3, Penelope-28, RTE-249, RETX-10, SINE-994, Tad1-8, Tx1-29, Vingi-3
8	Simple: 5	Sat-5

A total of 132 TAGs were extracted, out of which 100 and 32 TAGs were hypomethylated and hypermethylated, respectively (Table 4). SINE TEs were the most frequent, followed by Gypsy in hypomethylated TAGs. L1 and Gypsy TEs were more frequent in hypermethylated TAGs. In case of some TAGs, more than one and different type of TEs were found associated with a single TAG.

**TABLE 4:** Hypomethylated and hypermethylated TAGs along with the frequencies of TE subclasses.

Class and number of TEs in TAGs	Hypomethylated TAGs	Hypermethylated TAGs
DNA: 52	DNA-5, P-1, Crypton-1, Dada-1, CACTA-6, Harbinger-3, hAT-15, Helitron-5, ISL2EU-2, Mariner-3, MuDR-4, PiggyBac-1, Novosib-1, Polinton-3, Sola-1	CACTA-2, Harbinger-4, hAT-4, Helitron-1, ISL2EU-1, Mariner-1, MuDR-1
ERV: 23	ERV-1, ERV1-6, ERV2-4, ERV3-9	ERV1-4
Integrated virus: 1	Caulimovirus-1	0
Interspersed repeat: 2	2	0
LTR: 37	LTR-1, BEL-1, Copia-5, DIRS-2, Gypsy-20	Copia-2, Gypsy-6
Multicopy gene: 2	Multicopy_gene-2	0
Non-LTR: 82	Non-LTR-1, L1-16, L2-1, R1-1, R2-1, RTE-7, CR1-3, Jockey-1, Kiri-1, Nimb-1, SINE-44, Tx1-3	CR1-5, Rex1-1, I-2, L1-6, L2-2, Outcast-1, Penelope-1, RTE-2, SINE-2

### Association of DNA Methylation with miRNAs and their Target mRNAs

A total of eight methylation-regulated miRNAs were identified (Supplementary Table S3), out of which six miRNAs were targeting 44 mRNAs (transcribed from DEGs). The mode of action of miRNAs showed that 29 mRNAs were targeted by cleaving and 16 mRNAs were targeted by binding (Table 5). A total of five hypomethylation-regulated miRNAs were found positively correlated with 28 downregulated target DEGs (due to upregulation of miRNA) and one hypermethylation-regulated miRNA was positively correlated with one upregulated target DEG (due to downregulation of miRNA). The remaining 15 target DEGs did not show a positive correlation with methylation in miRNA genes.

**TABLE 5:** Target DEGs of miRNAs transcribed from DMRs along with their encoded proteins.

miRNAs	Log_2_FC of DMRs	Target DEGs	Log_2_FC of DEGs	Product of DEGs
bta-mir-2285cq	−2.16	ACTR3	−22.82	ARP3, actin related protein 3 homolog
bta-mir-12022	−2.37	ALG2	−6.53	ALG2, alpha-1,3/1,6-mannosyltransferase
bta-mir-2285cq	−2.16	ATP8A1	−6.45	ATPase phospholipid transporting 8A1
bta-mir-12022	−2.37	ATXN1	−6.18	ataxin 1
bta-mir-12022	−2.37	C7	−4.72	complement C7
bta-mir-12022	−2.37	DIS3L2	−4.41	DIS3 like 3′-5′ exoribonuclease 2
bta-mir-12022	−2.37	DLGAP4	−4.14	DLG-associated protein 4
bta-mir-12063	−2.61	DPY19L3	−4.05	dpy-19 like C-mannosyltransferase 3
bta-mir-12022	−2.37	ECM1	−4.017	extracellular matrix protein 1
bta-mir-2285cq	−2.16	EI24	−3.97	EI24, autophagy-associated transmembrane protein
bta-mir-12022	−2.37	EIF4E3	−3.93	eukaryotic translation initiation factor 4E family member 3
bta-mir-12063	−2.61	EPAS1	−3.8	endothelial PAS domain protein 1
bta-mir-2285cq	−2.16	ERAP1	−3.61	endoplasmic reticulum aminopeptidase 1
bta-mir-12063	−2.61	FAM227B	−3.53	family with sequence similarity 227 member B
bta-mir-12022	−2.37	FBXW7	−3.49	F-box and WD repeat domain containing 7
bta-mir-2285cq	−2.16	FOXK1	−3.35	forkhead box K1
bta-mir-2285cq	−2.16	GIMAP1	−3.22	GTPase, IMAP family member 1
bta-mir-12022	−2.37	GLYR1	−3.15	glyoxylate reductase 1 homolog
bta-mir-12063	−2.61	IKBKB	−3.05	inhibitor of nuclear factor kappa B kinase subunit beta
bta-miR-10161–5p	2.35	ITPR2	−2.85	inositol 1,4,5-trisphosphate receptor type 2
bta-mir-2285cq	−2.16	KIAA0232	−2.84	KIAA0232 ortholog
bta-miR-10161–5p	2.35	LOC102412044	−2.82	l-lactate dehydrogenase A chain
bta-mir-2285cq	−2.16	LOC102415248	−2.79	killer cell lectin-like receptor subfamily I member 1
bta-mir-2285cq	−2.16	LRP11	−2.58	LDL receptor related protein 11
bta-mir-11986	−2.76	LSMEM1	−2.37	leucine rich single-pass membrane protein 1
bta-miR-126–5p	−2.08	MAPKAP1	−2.28	mitogen-activated protein kinase associated protein 1
bta-mir-12063	−2.61	NAP1L1	−1.63	nucleosome assembly protein 1 like 1
bta-mir-12063	−2.61	NEXN	−1.43	nexilin F-actin binding protein
bta-mir-2285cq	−2.16	PLXNA2	1.54	plexin A2
bta-mir-12022	−2.37	PON1	1.75	paraoxonase 1
bta-mir-12063	−2.61	PPHLN1	1.9	periphilin 1
bta-mir-12022	−2.37	PPIL4	2.23	peptidylprolyl isomerase like 4
bta-mir-12022	−2.37	RCN1	2.56	reticulocalbin 1
bta-mir-12063	−2.61	RMC1	2.9	regulator of MON1-CCZ1
bta-mir-12063	−2.61	RRP1B	2.9	ribosomal RNA processing 1B
bta-mir-11986	−2.76	SCARB1	2.97	scavenger receptor class B member 1
bta-mir-12063	−2.61	SENP3	3.32	SUMO specific peptidase 3
bta-mir-2285cq	−2.16	SIN3A	3.68	SIN3 transcription regulator family member A
bta-mir-12063	−2.61	SYNE3	4.05	spectrin repeat containing nuclear envelope family member 3
bta-mir-12063	−2.61	TFEC	4.76	transcription factor EC
bta-mir-12022	−2.37	TNRC6A	5.31	trinucleotide repeat containing 6A
bta-mir-12063	−2.61	TNRC6B	5.64	trinucleotide repeat containing 6B
bta-miR-10161-5p	2.35	USP34	6.06	ubiquitin specific peptidase 34
bta-mir-12022	−2.37	ZRANB1	7.31	zinc finger RANBP2-type containing 1

### Association of DNA Methylation with lncRNA Genes and their Target mRNAs

A total 140 methylated lncRNA genes were found transcribing 284 lncRNAs, out of which nine lncRNAs from seven lncRNA genes were found targeting 209 mRNAs (transcribed from DEGs) encoding 126 proteins, 2 tRNAs, and 2 snRNAs (Table 6). While analyzing targets of methylation-regulated miRNAs in methylation-regulated lncRNAs, it was found that only one methylated miRNA (bta-miR-12022) was sequestering the activity of six other methylated lncRNAs by binding with them (Supplementary Table S4). These lncRNAs were found to have methylation in the genic regions (exon and intron).

**TABLE 6:** Target DEGs of lncRNAs transcribed from methylated genes.

Methylated lncRNA genes	Target DEGs	
LOC112583939, LOC112584670, LOC112585162, LOC112585197, LOC102413993, LOC112585597, LOC102408241	Proteins	PRELID3B, MICU3, LOC112577670, CABCOCO1, SENP6, RBM38, FAM219B, GXYLT2, DNAJC13, RIF1, BBS10, LOC112584770, LOC102415513, THNSL1, LOC102405919, CDK19, PTHLH, LOC102399155, FKBP3, STRN3, SEC11C, CENPU, PAXBP1, ATP5PF, BTG3, PFN2, UBE2G2, NCKAP1, CD302, EPC2, SUZ12, PPM1D, SAP30, HPGD, PLGRKT, PLEKHA5, AMN1, LOC102406990, PTGES3, SLC25A16, PCBD1, LOC102399263, CNIH4, TMEM262, TIPRL, HSD17B7, ZNF644, NEXN, SRSF11, SPATA6, ATPAF1, RRAGC, PI4K2B, ZNRF2, SRI, SLC25A46, FAM174A, RNF130, MPC1, QKI, CD24, SLIRP, FAM214A, PIGB, TPM1, AP3S1, RPS27A, VRK2, GEN1, GPR180, NDFIP2, PAN3, WFDC2, SYS1, COMMD7, HACD1, TRDMT1, IMPAD1, NSMCE2, FDX1, FAM76B, AASDHPPT, ISCU, FGF2, CBFB, EIF5, FAM177A1, RCN2, BTBD1, CMC1, LOC102402381, TXNL1, C22H18orf54, MBD2, RNF138, UBE2D1, KDELR2, NTAN1, DEXI, TTC14, KPNA4, SLC35B3, AGPS, RHOT1, ETNK1, FAM118B, RIIAD1, PTGR2, RPL31, PCSK7, STX2, KLC1, G2E3, PXK, FAM210A, DMD, RAB5A, ERP44, IVNS1ABP, SH2D1B, CCDC18, MKLN1, MAPKAP1, ZNF26, PPP1R37, GDNF
	snRNAs	LOC112578276, LOC112581316
		tRNAs	TRNAN-GUU, TRNAY-AUA

1 https://github.com/agordon/fastx_toolkit.

2 https://www.ncbi.nlm.nih.gov/assembly/GCF_003121395.1/.

### Development of the Web Resource, BSCM2TDb

**FIGURE 6:**
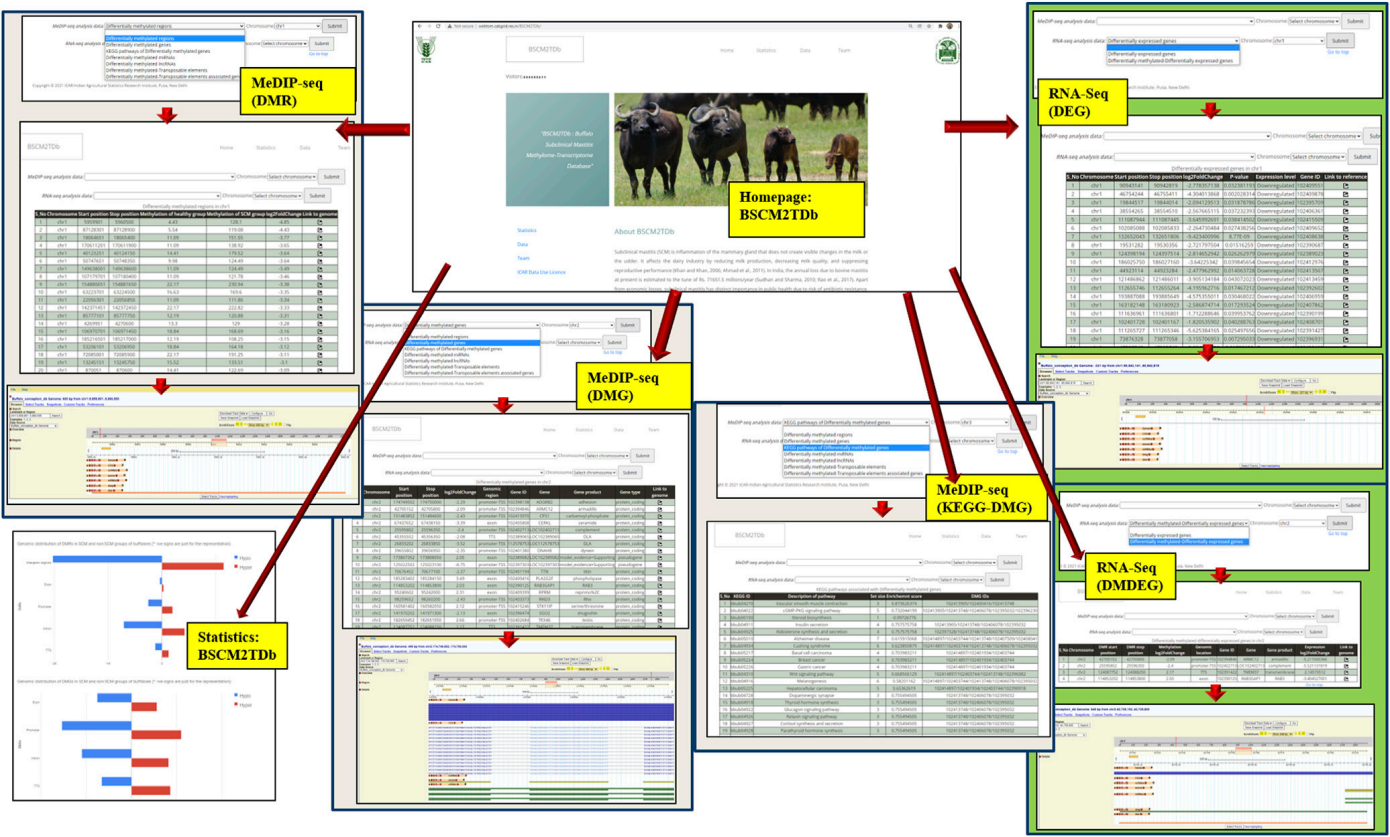
Layout of the web resource BSCM2TDb.

The BSCM2TDb web resource has four main tabs, namely, Home, Statistics, Data, and Team (Figure 6). The *Home* page has a brief introduction about the database. The navigation key *Statistics* included a pie chart, showing the proportion of all included data, i.e., 7,900 DMRs, 370 DMGs, 208 DMG-KEGG pathways, 8 DM-miRNAs, 138 DM-lncRNAs, 3,377 DM-TEs, 131 DM-TAGs, 4,638 DEGs, and 64 DM-DEGs. The *Data* page is the main analyses result page that provides the options in the drop-down menu to navigate to the complete table of selected option. The *Team* page included the team member’s name and link to the profile page of each member.

## Discussion

SCM is an inflammatory disease in water buffaloes that causes major losses to the dairy industry. The present study is the first genome-wide DNA methylation (MeDIP-Seq) study to compare an SCM group with a healthy group to understand the regulation of genes involving DNA methylation during host–pathogen interaction specific to SCM in buffaloes. MeDIP-Seq is a popular technique for the extraction of genome-wide DNA methylation, which has been used in numerous SCM studies on mammals including bovine (Song et al., 2016; Chen et al., 2019). A high correlation was found among MeDIP-Seq libraries, which is due to the sample taken from the buffaloes of the same genotype. The average methylation in healthy buffaloes was higher than that in SCM-infected buffaloes, which suggests that the upregulation and activation of certain genes may be required for the development of SCM. DNA methylation was found mostly in the intergenic region followed by intronic region in both healthy control and SCM groups, which is in agreement with other studies as well (Song et al., 2016; Fang et al., 2017; Wang et al., 2020). The DNA methylation level was significantly higher in the gene body than in upstream and downstream regions of genes; similar results were found by Wang et al. (2020).

We observed more hypomethylation in the SCM group than in the control group, which is in concordance with the results found in neutrophils of *E. coli* mastitis cows (Ju et al., 2020) and further suggests that the upregulation of certain genes may be required for the development of SCM during host–pathogen interaction. The 3,950 DMRs identified were annotated to 370 DMGs, and most of the DMGs were enriched in the promoter region, which is in concordance with earlier reports (Irizarry et al., 2009; Song et al., 2016). Some DMGs were found related to the immune system like colony-stimulating factor 2 receptor beta common subunit (CSF2RB) and granulocyte-macrophage colony-stimulating factor receptor subunit alpha-like (LOC102408349), which are cytokines considered essential for the survival, proliferation, and differentiation of blood cells such as granulocyte and macrophages as reported by Jeong et al. (2014). Additionally, antigen WC1.1-like (LOC102391350) expresses on subsets of CD4^−^CD8^−^ gamma delta T lymphocytes (Wijngaard et al., 1994), and V-set domain-containing T-cell activation inhibitor 1 (VTCN1) negatively regulates T-cell-mediated immune response. The DLA class II histocompatibility antigen DR-1 beta chain-like (LOC102389065) involves in the adaptive immune response. From results of the KEGG pathway analysis, interestingly, it was found that the *S. aureus* infection pathway was found significantly activated due to hypomethylation, suggesting that a major causal pathogen for SCM in this study could also be *S. aureus* and that there is a possible regulatory role of DMGs in the host response to *S. aureus*-induced mastitis, which is in agreement with the previous study involving SCM in cows (Wang et al., 2020). Additionally, important pathways activated due to hypomethylation related to immunity were Th17 cell differentiation and antigen processing and presentation, along with activation of pathways related to defense response such as Fc gamma R-mediated phagocytosis, phagosome, and leukocyte transendothelial migration. There is an important role of Th17 cell differentiation in the immune regulation of T cells (Sordillo, 2011) and *S. aureus* mastitis (Zhao et al., 2015; Wang et al., 2020). In the present study, Th17 cell differentiation pathway activation in response to SCM is further confirmation of the significance of Th17 cells in host immune response and regulation mediated by DNA methylation during SCM. The activated KEGG pathway related to cell adhesion molecules confirms previous studies involving the role of methylation in cell adhesion, which influences immune cell function during host–pathogen interaction (Li et al., 2018; Wang et al., 2020).

Furthermore, DNA methylation was compared with transcriptome data in the present study to understand the effect of DNA methylation on gene expression in response to SCM in buffaloes. A total of 4,778 significant DEGs were extracted specific to SCM, out of which 67 genes were also found differentially methylated along with differentially expressed, suggesting that DNA methylation could be one of the epigenetic regulatory mechanisms of gene expression during SCM development. Among these genes, 73% DMGs were negatively correlated with gene expression in the promoter region while the rest were positively correlated with gene expression, which is in agreement with the previous studies (Selamat et al., 2012; Song et al., 2016), suggesting dynamic regulation due to DNA methylation in response to SCM. Some of the important DMGs with correlated differential gene expression were MED25, BLOC1S6, CREB3L1, FRY, protein kinase C-binding protein 1-like, VPS11, CSF2RB, granulocyte-macrophage colony-stimulating factor receptor subunit alpha-like (LOC102408349), C3 and PZP like, alpha-2-macroglobulin domain containing 8 (CPAMD8), and MYSM1, which could be involved in the development of SCM during host–pathogen interaction in buffaloes. Out of these genes, CSF2RB, LOC102408349, C3 and PZP like, and CPAMD8 were found to be downregulated due to hypermethylation in the promoter region during the development of SCM by suppressing immune response in buffaloes, which are known to be involved in immune response.

Additionally, a large number of TEs were found in DMRs in response to SCM in the present study. Apart from TEs in intergenic and intronic regions, there were higher hypomethylated TEs in the promoter region too, which is in agreement with the previous studies involving DNA methylation in cattle tissues (Zhou et al., 2020). The most common class of TEs in DMRs and TAGs was of the SINE family. A large number of TEs in differentially methylated intergenic and intronic regions suggests a role of DNA methylation in the stability of the genome (Adelson et al., 2009; Iwasaki et al., 2015; Czech and Hannon, 2016) and more hypomethylated TEs in response to SCM suggests more activation of TEs during the host–pathogen interaction during SCM. Similarly, probable differentially methylated TAGs were also found more hypomethylated in response to SCM.

Interestingly, we also studied the DNA methylation within miRNA and lncRNA transcribing genes to understand the indirect regulation of gene expression through DNA methylation at the posttranscriptional level. These methylation-regulated lncRNAs and miRNAs were found to target mRNAs transcribed through DEGs specific to SCM, suggesting that the DNA methylation is affecting the expression of genes not only directly at the transcription level but also indirectly at the posttranscriptional level during host–pathogen interaction specific to SCM in buffaloes, which is in agreement of with the findings of Lim et al. (2017) and Saripalli et al. (2020) that the correlation between DNA methylation and gene expression is nonlinear and complex. Similar findings were found by Ju et al. (2020) in the case of *E. coli*-infected mastitis cows. It is also reported that miRNA plays an important regulatory role in immune and inflammatory responses to mastitis in cows (Ju et al., 2019) and DNA methylation plays a role in the regulation of around 10% miRNAs (Han et al., 2007).

All the results obtained from the present study were compiled and catalogued in the form of a web resource, BSCM2TDb. The present finding can not only be used for understanding the molecular regulation of mastitis disease but can also be used to identify candidate epigenetic markers related to the disease (Zheng et al., 2018). Such combined analysis of DNA methylome and transcriptome map has already been successfully used to identify candidate genes of mastitis disease susceptibility in cattle (Song et al., 2016). The unavailability of web resources related to mastitis disease obviates the development of BSCM2TDb, which catalogues the results obtained from this study at a single place for easy access. This will be useful to the bovine scientific community to be utilized in further studies and research.

## Conclusion

The study is based on lactating Indian Murrah water buffaloes from the ICAR-CIRB, India, farm for the incidence of SCM. Here, DNA methylation was compared with transcriptome data to understand its effect on gene expression in response to SCM. The genomic annotation of obtained peaks is abundant in the intergenic region. On comparison of methylation in the SCM group vs control group, out of the total 3,950 DMRs, 2,451 were hypomethylated, while 1,449 were hypermethylated. Out of these 3,950 DMRs, 370 DMGs (169 hypermethylated and 201 and hypomethylated) were extracted. While annotating the 21,028 DERs with buffalo RefSeq annotation data, 4,778 DEGs (2,908 downregulated and 1,870 upregulated) were obtained. The KEGG pathway enrichment analysis revealed 142 pathways for 367 DMGs. A comparison of DMGs from MeDIP-Seq analysis and DEGs from RNA-Seq analysis shows that 67 DMGs have differential expression as well. Furthermore, 33 DMGs had gene expression as per methylation. Analyses also resulted in 3,377 TEs within DMRs and 132 TAGs. The CSF2RB, LOC102408349, C3 and PZP like, and CPAMD8 genes, which are known to involved in immune response, were found to be downregulated due to hypermethylation in the promoter region in our study. The present study is the first genome-wide DNA methylation study specific to SCM in buffaloes of the Murrah breed, aiming to understand the role of epigenetic regulation involving DNA methylation of genes involved in host–pathogen interaction during SCM in buffaloes. Interestingly, the present study also sheds a brief light to the role of DNA methylation in indirect regulation of SCM-specific mRNAs at the posttranscriptional level by methylation-regulated miRNAs and lncRNAs. All this information has been catalogued at one place in the BSCM2TDb, which may be of immense use to buffalo researchers in the endeavor of mastitis control and management for higher milk production.

## Data Availability

The following data of whole-genome DNA methylation and transcriptome data of buffalo are submitted in the NCBI repository with BioProject ID PRJNA739886; MeDIP-Seq SRA IDs SRR14879252, SRR14879253, SRR14879254, SRR14879255, SRR14879256, SRR14879257, SRR14879258, SRR14879262, SRR14879272, SRR14879273, SRR14879274; RAN-Seq SRA IDs SRR14879259, SRR14879260, SRR14879261, SRR14879263, SRR14879264, SRR14879265, SRR14879266, SRR14879267, SRR14879268, SRR14879269, SRR14879270, SRR14879271.
