# Supplementary figures and images for: Genome-Wide DNA Methylation and Its Effect on Gene Expression During Subclinical Mastitis in Water Buffalo
Source: Front Genet. 2022 Mar 15;13:828292. doi: 10.3389/fgene.2022.828292 (PMC8965078; doi:10.3389/fgene.2022.828292)

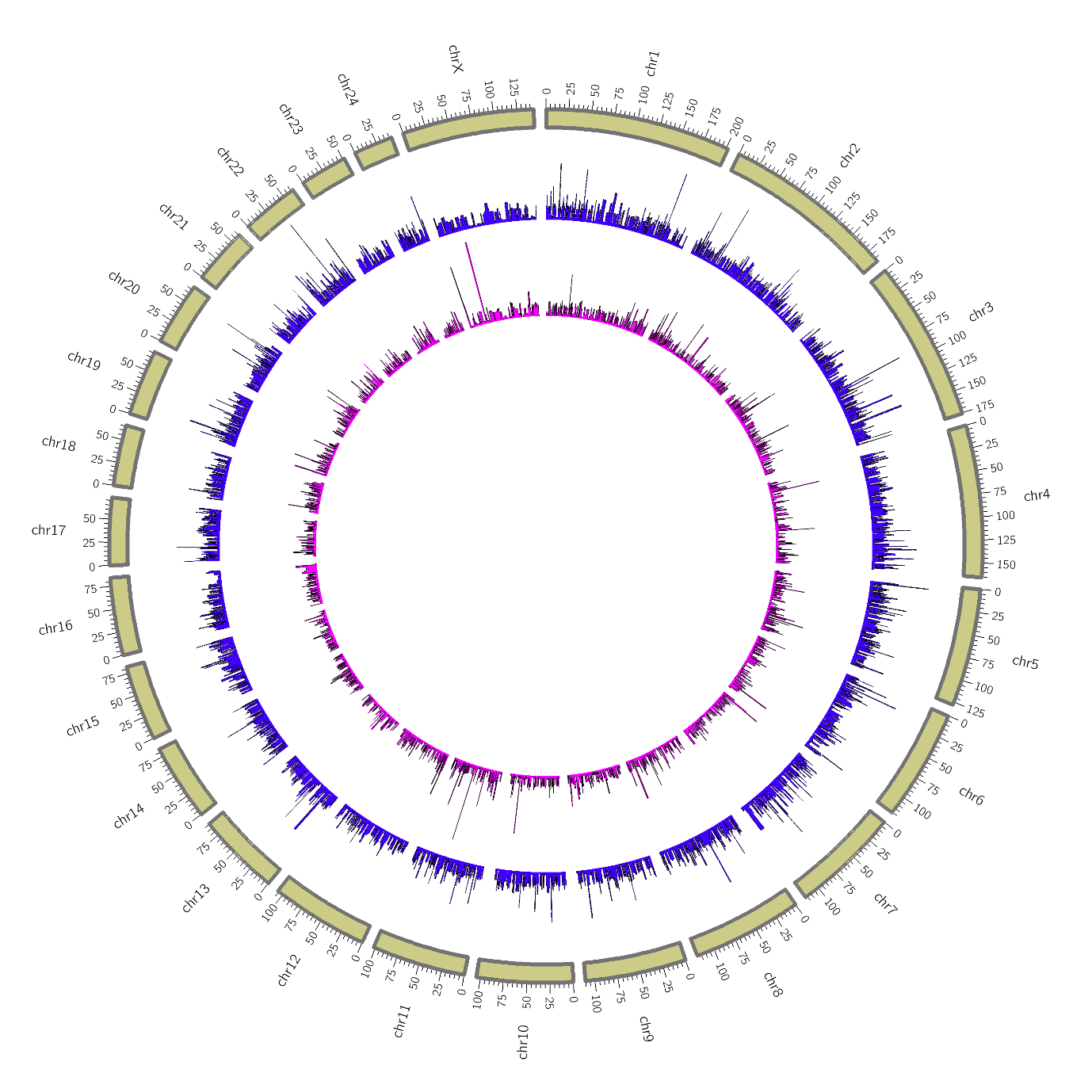

Supplement: Supplementary file 1 [file Image3.TIF]

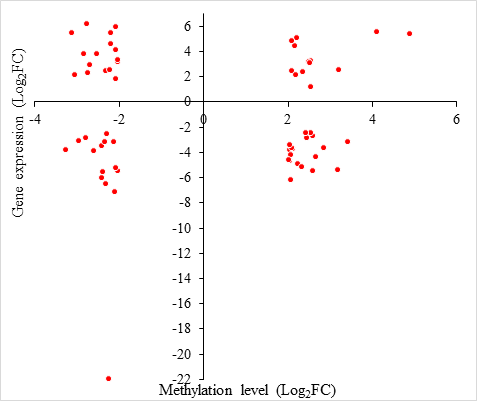

Supplement: Supplementary file 2 [file Image4.TIF]

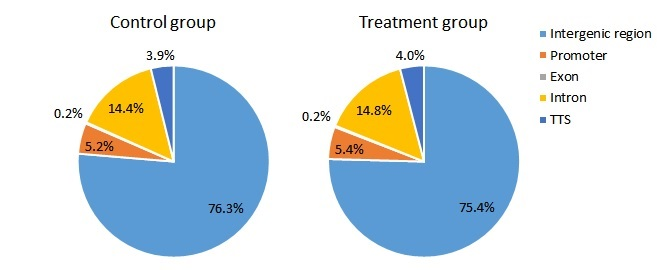

Supplement: Supplementary file 3 [file Image2.TIF]

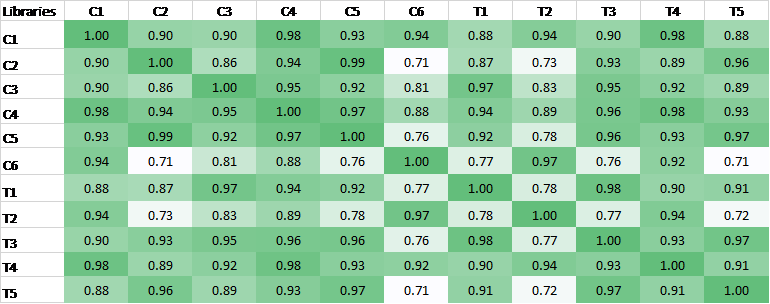

Supplement: Supplementary file 4 [file Image1.TIF]
