# Supplementary material for: Genome-Wide DNA Methylation and Its Effect on Gene Expression During Subclinical Mastitis in Water Buffalo
Source: Front Genet. 2022 Mar 15;13:828292. doi: 10.3389/fgene.2022.828292 (PMC8965078; doi:10.3389/fgene.2022.828292)
Supplement: Supplementary file 5 [file DataSheet1.docx]

**Genome-wide DNA methylation and its effect on gene expression during subclinical mastitis in water buffalo**

Varij Nayan^1,#^, Kalpana Singh^2,#^, Mir Asif Iquebal^2,#^, Sarika Jaiswal^2^, Anuradha Bhardwaj^3^, Chhama Singh^1^, Tanvi Bhatia^1^, Sunil Kumar^1^, Rakshita Singh^1^, M.N. Swaroop^1^, Rajesh Kumar^1^, S.K. Phulia^1^, Anurag Bharadwaj^1^, T.K. Datta^1^ , Anil Rai^2^ , Dinesh Kumar^2,*^,

^1^ICAR-Central Institute for Research on Buffaloes (CIRB), Molecular Endocrinology, Functional Genomics & Computational Biology Laboratory, Animal Biochemistry, APR Division, Hisar-125001, Haryana (India)

^2^Centre for Agricultural Bioinformatics, ICAR-Indian Agricultural Statistical Research Institute (IASRI), Pusa, New Delhi-110012, Delhi (India)

^3^ICAR-National Research Centre on Equines (NRCE), Hisar-125001, Haryana (India)

**Supplementary Tables**

**Supplementary Table 1.** Number of reads, GC% and % alignment of MeDIP-seq and RNA-seq libraries.

| **Library*** | **PE reads in MeDIP-seq** | **%GC in MeDIP-seq** | **%alignment in MeDIP-seq** | **PE reads in RNA-seq** | **%alignment in RNA-seq** |
| --- | --- | --- | --- | --- | --- |
| C1 | 33317636 | 47 | 70.99 | 27972539 | 90.01 |
| C2 | 43857760 | 44 | 72.94 | 29144050 | 89.79 |
| C3 | 28020385 | 47 | 69.83 | 28000000 | 89.07 |
| C4 | 33308576 | 46 | 71.09 | 29368194 | 89.21 |
| C5 | 38578585 | 45 | 72.19 | 29532068 | 91.54 |
| C6 | 28047588 | 49 | 69.45 | 31745193 | 87.19 |
| SCM1 | 30491748 | 46 | 70.35 | 24546924 & 29292837 | 77.18 & 81.56 |
| SCM2 | 28386890 | 49 | 69.79 | 39335934 | 53.83 |
| SCM3 | 34176035 | 46 | 71.07 | 25715376 | 49.62 |
| SCM4 | 32772781 | 47 | 70.87 | 33462740 | 55.21 |
| SCM5 | 41544586 | 46 | 72.15 | 30303602 | 53.53 |

*C1-C6: Control group and SCM1-SCM5: Subclinical mastitis group

**Supplementary Table 2.** Peaks and their genomic annotation into intergenic, promoter, intron, exon, and TTS regions in the libraries

| **Library** | **Total peaks** | **Peaks on unknown chromosome** | **Intergenic region** | **Promoter** | **Exon** | **Intron** | **TTS** |
| --- | --- | --- | --- | --- | --- | --- | --- |
| C1 | 28751 | 386 | 24440 | 1508 | 69 | 4430 | 1149 |
| C2 | 47941 | 263 | 37749 | 2400 | 88 | 5822 | 1749 |
| C3 | 23625 | 244 | 17154 | 1315 | 49 | 3977 | 1020 |
| C4 | 30407 | 281 | 22785 | 1623 | 68 | 4619 | 1196 |
| C5 | 40355 | 306 | 31094 | 2094 | 86 | 5409 | 1544 |
| C6 | 18395 | 197 | 13126 | 996 | 45 | 3410 | 728 |
| SCM1 | 24701 | 243 | 17787 | 1474 | 56 | 4229 | 1045 |
| SCM2 | 19683 | 251 | 14152 | 1148 | 109 | 3487 | 779 |
| SCM3 | 33362 | 288 | 25205 | 1790 | 68 | 4868 | 1320 |
| SCM4 | 29682 | 248 | 22195 | 1622 | 58 | 4489 | 1212 |
| SCM5 | 47375 | 385 | 37063 | 2376 | 87 | 5828 | 1883 |

**Supplementary Table 3**. Details of miRNA transcribed from DMRs along with the pre-miRNA sequences

| **miRNA** | **Sequence** | **Pre-miRNA sequence** | **Position** | **Log_2_FC of DMR** |
| --- | --- | --- | --- | --- |
| bta-miR-126-5p | cauuauuacuuuugguacgcg | UGACGGGACAUUAUUACUUUUGGUACGCGCUGUGACACUUCAAACUCGUACCGUGAGUAAUAAUGCGCUGUCA | 09-29 | -2.08 |
| bta-miR-126-3p | cguaccgugaguaauaaugcg | UGACGGGACAUUAUUACUUUUGGUACGCGCUGUGACACUUCAAACUCGUACCGUGAGUAAUAAUGCGCUGUCA | 47-67 | -2.08 |
| bta-mir-4286 | accccacuccugguacc | AAUUGAGAAGGGAUAAGGAGGUGUGUGGAGAAGGCAAUGGCACCCCACUCCUGGUACCAACAU | 42-58 | -2.76 |
| bta-miR-10161-5p | guggagucagacauacugaguga | GUAUUAGUCUGUGGAGUCAGACAUACUGAGUGACUUUCACUUCAGUUCAGUUAAGUUCAGUCACUCAGUCGUGUCCGACUCUUUGCUACCCC | 11-33 | 2.35 |
| bta-mir-11986 | uuguccaaugaguuaguccuu | UUGUCCAAUGAGUUAGUCCUUCACAUCAGGUGGCCAAAGUAUUGGAGUUUCAGC | 01-21 | 2.21 |
| bta-mir-2285cq | aaaaguuuguuccaguuuuucu | AAAAGUUUGUUCCAGUUUUUCUAUCACAUCUUAUGGAAAAACUGGAAUGAACUUUUUGG | 01-22 | -2.16 |
| bta-mir-12022 | uuggacacgacaagugacuuu | UUGGACACGACAAGUGACUUUCGCUUAUGUUAAAGUGAAGUCGCUCAGUCGUGUCCAACU | 01-21 | -2.37 |
| bta-mir-12063 | acugguccaggaagauucu | AACUUCCCUGGUGGUCCAGUAGUUAAGAAUCCGCCUGCCAAGGCAGGGGACACGGGUUCAGUCACUGGUCCAGGAAGAUUCU | 64-82 | -2.61 |

**Supplementary Table 4**. Target methylated lncRNAs of methylated miRNA (bta-mir-12022) along with their level of methylation

| **Target lncRNAs of bta-mir-12022**  **(methylation log_2_FC= -2.37)** | **Multiplicity** | **Log_2_FC of DMRs** |
| --- | --- | --- |
| LOC102396368 | 1 | 2.11 |
| LOC112583719 | 1 | -2.53 |
| LOC112579925 | 2 | -2.06 |
| LOC112584700 | 2 | -2.05 |
| LOC102396291 | 3 | 2.78 |
| LOC112586803 | 1 | -2.01 |
